# Characterization of the B-Cell Epitopes of *Echinococcus granulosus* Histones H4 and H2A Recognized by Sera From Patients With Liver Cysts

**DOI:** 10.3389/fcimb.2022.901994

**Published:** 2022-06-13

**Authors:** Andrea Maglioco, Facundo A. Agüero, María Pía Valacco, Alejandra Juárez Valdez, Margot Paulino, Alicia G. Fuchs

**Affiliations:** ^1^ Universidad Abierta Interamericana (UAI), Centro de Altos Estudios en Ciencias Humanas y de la Salud (CAECIHS), Buenos Aires, Argentina; ^2^ Consejo Nacional de Investigaciones Científicas y Técnicas (CONICET), Buenos Aires, Argentina; ^3^ Centro de Estudios Químicos y Biológicos por Espectrometría de Masas (CEQUIBIEM), Instituto de Química Biológica Ciencias Exactas y Naturales- Consejo Nacional de Investigaciones Científicas y Técnicas (IQUIBICEN-CONICET), Facultad de Ciencias Exactas y Naturales- Universidad de Buenos Aires (UBA), Buenos Aires, Argentina; ^4^ Departamento de Experimentación y Teoría de la Estructura de la Materia y sus Aplicaciones, Facultad de Química, Bioinformatica DETEMA- Udelar, Universidad de la República, Montevideo, Uruguay; ^5^ Instituto Nacional de Parasitología “Dr Mario Fatala- Chaben”, (Administración Nacional de Laboratorios e Institutos de Salud )ANLIS‐Malbrán, Buenos Aires, Argentina

**Keywords:** Histones, *Echinococcus granulosus*, epitopes, cell extract, extracellular

## Abstract

Cystic echinococcosis (CE) is a zoonotic disease worldwide distributed, caused by the cestode *Echinococcus granulosus* sensu lato (*E. granulosus*), with an incidence rate of 50/100,000 person/year and a high prevalence in humans of 5-10%. Serology has variable sensitivity and specificity and low predictive values. Antigens used are from the hydatid fluid and recombinant antigens have not demonstrated superiority over hydatid fluid. A cell line called EGPE was obtained from *E. granulosus* sensu lato G1 strain from bovine liver. Serum from CE patients recognizes protein extracts from EGPE cells with higher sensitivity than protein extracts from hydatid fluid. In the present study, EGPE cell protein extracts and supernatants from cell colonies were eluted from a protein G affinity column performed with sera from 11 CE patients. LC-MS/MS proteomic analysis of the eluted proteins identified four *E. granulosus* histones: one histone H4 in the cell extract and supernatant, one histone H2A only in the cell extract, and two histones H2A only in the supernatant. This differential distribution of histones could reflect different parasite viability stages regarding their role in gene transcription and silencing and could interact with host cells. Bioinformatics tools characterized the linear and conformational epitopes involved in antibody recognition. The three-dimensional structure of each histone was obtained by molecular modeling and validated by molecular dynamics simulation and PCR confirmed the presence of the epitopes in the parasite genome. The three histones H2A were very different and had a less conserved sequence than the histone H4. Comparison of the histones of *E. granulosus* with those of other organisms showed exclusive regions for *E. granulosus*. Since histones play a role in the host-parasite relationship they could be good candidates to improve the predictive value of serology in CE.

## 1 Introduction

Cystic echinococcosis (CE) is a zoonotic disease worldwide distributed, caused by the cestode *Echinococcus granulosus* sensu lato, with an incidence rate of 50/100,000 person/year and a high prevalence in humans of 5-10%. In Latin American countries, it is an endemic disease with active transmission, with a proportion of infected young people that reaches 15% (Larrieu et al., 2019). In Argentina, 630 CE cases were confirmed in 2018-2019 (in a period of 48 weeks)^1^ and 12.1% out of the 479 new cases confirmed in Buenos Aires province in 2014-2016 were younger than 18 years old (Álvarez et al., 2018). Based on the latest expert consensus on cystic echinococcosis four genotypes clusters have been demonstrated for *E. granulosus* s.l. including: sensu stricto (G1/3), *E. equinus* (G4), *E. ortleppi* (G5) and *E. canadensis* (G6-8/10) (Vuitton et al., 2020). In Argentina, *E. granulosus* sensu stricto (G1/3) has the highest prevalence in both patients and livestock (Cucher et al., 2016).

The parasite has a complex life cycle involving two hosts. The hermaphrodite worm is developed in the intestinal lumen of canids, which are the definitive hosts. Then, fertile proglottids containing oncospheres are shed into the soil by feces, and the intermediate hosts, ungulate animals, or aberrant hosts such as humans or cats (Avila et al., 2021), acquire the parasite *per os*. The cycle closes when canids become infected by eating visceral organs from infected ungulate animals. In the intermediate host, oncospheres come into the abdominal cavity through the intestinal wall after activation, and colonize visceral organs. In humans, the liver has the highest frequency (80%) of infection, followed by the lung (15%) and other organs. The parasitic infection causes high morbidity mainly when it is located in the bone or central nervous system (3-5%). The larva or metacestode, which develops in the intermediate host, has an asexual reproduction, forming protoscoleces from the germinal layer, which is the most internal layer in contact with the cyst cavity full of hydatid fluid. This fluid contains protoscoleces, cells, salts, proteins, and amino acids, and its composition changes according to metacestode viability (Ahn et al., 2015). The germinal layer is covered by the laminar layer, which is the parasite outer acellular layer that participates in the host-parasite interchange. The laminar layer could be damaged by trauma, cyst growth or complications, a fact that leads the protoscoleces to spill in the host body, colonizing other organs.

The clinical suspicion of CE is based mainly on epidemiological data and symptoms. The infection is then confirmed by typical images, which allow disease staging; serology helps to confirm the imaging diagnosis (Zait and Hamrioui, 2020) and analysis of parasite material constitutes the *gold* standard (Reinehr et al., 2020; Pena et al., 2021). The serology methods, standardized in each laboratory, have variable sensitivity and specificity due to cross-reactions or weak antigen recognition and thus, low predictive values. Serology for differential CE diagnosis and infection follow-up is a research field in progress, with new laboratory methods such as Raman spectroscopy to evaluate serum samples (Yue et al., 2020) and the proposal of new recombinant antigens, initially examined in infected livestock (Liang et al., 2020). However, no recombinant antigen has demonstrated superiority over hydatid fluid extract for CE diagnosis, and serology is not a useful method for infection or treatment follow-up in humans (Sánchez-Ovejero et al., 2020).

In our laboratory, a cell line called EGPE was obtained from *E. granulosus* sensu lato G1 strain from bovine liver (Echeverría et al., 2010). By using this cell line in a paired case-control study, we have previously found that serum from CE patients recognizes protein extracts from EGPE cells at two growth stages with higher sensitivity than those extracted from hydatid fluid (Maglioco et al., 2019).

Histones are proteins associated with cell cycle regulation, protein synthesis and DNA repair. In *E. granulosus*, histones have been found in the nucleosome and other subcellular localizations of protoscoleces (Lorenzatto et al., 2015). Histones H2A, H2B, H3 and H4 are core histones assembled into an octamer around DNA, forming a nucleosome. They are basic proteins divided into two classes: lysine-rich (H1, H2A and H2B) and arginine-rich (H3 and H4) (DeLange and Smith, 1971), sharing a similar structure: three central α-helices connected by loops on C-terminal and N-terminal end. The N-terminus is the site where post-translational modifications, such as acetylation, methylation, citrullination, ubiquitination, phosphorylation and SUMOylation, occur. Histone epigenetic modification is triggered by autocrine factors and parasite-host interactions (Magalhães et al., 2020). Moreover, histone overexpression could be involved in the parasite response to injury (Singh et al., 2010). Histones are encoded by different multi-variable genes and variability contributes to chromatin regulation (Ferrand et al., 2020). Canonical histones are synthesized in the S phase of the cellular cycle, and histone variants substitute for H4, H3, H2A, H2B and H1 confer structural and functional features, and are synthesized independently of the cell cycle, having a single-copy gene (Singh et al., 2010).

In CE, detection of reactive antibodies against *E. granulosus* metacestode antigenic proteins varies depending on the parasite localization. Serological tests have revealed differences in sensitivity whether the parasite is localized in the liver or lungs^2^. Serology specificity also varies because *E. granulosus* shares antigens with other parasites such as *Taenia solium*, *Fasciola hepatica* and *E. multilocularis* (Rassy et al., 2010).

To improve CE serology, we chose the histone family, among the antigenic proteins recognized by sera from CE patients only in the liver. Histones were chosen due to their intra- and extracellular localization and for their role in the naïve immune response. The histones identified were characterized and bioinformatics molecular studies of the epitopes were performed. PCR and sequencing were used to confirm the epitope identification in the *E. granulosus* sensu stricto G1 strain.

## 2 Materials and Methods

### 2.1 Ethics Statement and Serum Samples

Serum samples from CE patients were obtained by Dr. Jorge Gentile from Hospital Municipal Ramón Santamarina, Tandil, Argentina. Sera from patients with cysticercosis and fascioliasis were donated by Dr. Elizabeth Luz Sánchez Romaní, Laboratorio de Zoonosis Parasitaria CNSP-INS-Perú. All protocols and procedures were approved by the Ethics Committee of the ‘Universidad Abierta Interamericana’, Buenos Aires, Argentina (number 01011). Patient serum samples were from an anonymized laboratory serum stock (Maglioco et al., 2019).

### 2.2 EGPE Cell Culture, Protein Extracts, and Supernatant

EGPE is a cell line obtained from bovine *E. granulosus* pe G1 maintained at our laboratory. Cells from passages 35 to 40 were used for all experiments. Briefly, EGPE cells were grown in medium 199 (Sigma), 1 mM sodium pyruvate (sodium salt, extra pure, Anhedra, Beijing, China) and 78 μg/mL β-mercaptoethanol (Merck, Darmstadt, Germany) at pH 7.9 (37°C; CO_2_:air; 5/95%) and EGPE cell colonies were performed in 2% agarose (20,000 cells/well) (Echeverría et al., 2010). Cells were grown in a liquid medium for 20 days and protein extracts were obtained as previously (Maglioco et al., 2019). Briefly, cells were washed five times with DPBS and incubated in lysis buffer (8 mmol/L CHAPS,MP Biomedicals, 10 mmol/L Tris –HCl, pH 8, 2 mmol/L EDTA, 0.1% B‐mercaptoethanol,MP Biomedicals, and 1/100 protease inhibitor cocktail, Sigma‐Aldrich), at 4°C for 2 hours. Samples were then frozen-thawed three times and spun down at 10 000 g.

Cell colony supernatants were obtained after 5 days of incubation. Debris was removed by centrifuging the supernatants three times (3000 rpm). Then, samples were stored in aliquots at -20°C until use.

### 2.3 Protein Identification

#### 2.3.1 Extraction of Protein Fraction

EGPE cell protein extracts were first passed through a gel filtration column (1.6 cm x 90 cm, Sephacryl S-200 HR GE Healthcare). Protein fractions were identified by absorbance (205-280 nm) in a spectrophotometer (Biowave II+ , Biochrome Ltd., Cambridge, England). Every fraction with higher absorbance was concentrated through a 3K cut‐off membrane concentrator (Pierce, Thermo Scientific). The reactivity of these fractions and that of the supernatant of EGPE colonies was analyzed by Western blot (Maglioco et al., 2019) by using a pool of sera from 11 patients with CE with only hepatic localization (**Supplementary Figure 1**). Then, reactive protein fractions were passed through an affinity column (Protein G HP SpinTrap - GE Healthcare) prepared with this pool of CE sera (cases) or through an affinity column performed with a pool of sera from two patients with cysticercosis and two patients with fascioliasis (controls). Eluted proteins were concentrated through a 3K cut‐off membrane concentrator (Pierce, Thermo Scientific). A 15% SDS–PAGE was performed to concentrate and clean-up protein extracts prior to in-gel digestion (data not shown).

#### 2.3.2 Protein Digestion and Mass Spectrometry Analysis

Proteins were then digested and analyzed by Mass Spectrometry Analysis at the Proteomics Core Facility of the CEQUIBIEM, Faculty of Exact Sciences, University of Buenos Aires/IQUIBICEN CONICET, National Research Council, Argentina. Protein bands excised from Coomassie blue-stained SDS-PAGE gels were sequentially washed and destained with 50 mM ammonium bicarbonate, 25 mM ammonium bicarbonate, 50% acetonitrile, and 100% acetonitrile, and then reduced and alkylated with 10 mM dithiothreitol and 20 mM iodoacetamide and in-gel digested with 100 ng Trypsin (Promega V5111) in 25 mM ammonium bicarbonate overnight at 37°C. Peptides were recovered by elution with 50% acetonitrile-0.5% trifluoroacetic acid, including brief sonication, and further concentrated by speed-vacuum drying. Samples were resuspended in 15 μL of water containing 0.1% formic acid, desalted using C18 zip tips (Merck Millipore) and eluted in 10 μL of water: acetonitrile: formic acid 40:60:0.1%. The digests were analyzed by nanoLC-MS/MS in a Thermo Scientific QExactive Mass Spectrometer coupled with a nanoHPLC EASY-nLC 1000 (Thermo Scientific). For LC-MS/MS analysis, approximately 2 μg of peptides was loaded onto a reverse-phase column (C18, 2 µm, 100A, 50 µm x 150 mm) Easy-Spray Column PepMap RSLC (P/N ES801) suitable to separate protein complexes with a high degree of resolution. The flow rate used for the nano-column was 300 nL min-1 and the solvent range from 7% B (5 min) to 35% (120 min). Solvent A was 0.1% formic acid in water, whereas solvent B was 0.1% formic acid in acetonitrile. The injection volume was 2 µL. A voltage of 3.5 kV was used for Electro Spray Ionization (Thermo Scientific, EASY-SPRAY).

XCalibur 3.0.63 (Thermo Scientific) software was used for data acquisition. Full-scan mass spectra were acquired in an Orbitrap analyzer. The scanned mass range was 400-1800 m/z, at a resolution of 70000 at 400 m/z, and the twelve most intense ions in each cycle were sequentially isolated, fragmented by higher-energy collision dissociation, and measured in an Orbitrap analyzer. Peptides with a charge of +1 or with an unassigned charge state were excluded from fragmentation for MS2.

QExactive raw data were processed using the Proteome Discoverer software (version 2.1.1.21, Thermo Scientific) and searched against the *E. granulosus* sequence database with trypsin specificity and a maximum of one missed cleavage per peptide. Carbamidomethylation of cysteine residues was set as a fixed modification and oxidation of methionine was set as variable modification. Proteome Discoverer searches were performed with a precursor mass tolerance of 10 ppm and product ion tolerance of 0.05 Da. Protein hits were filtered for high-confidence peptide matches with a maximum protein and peptide false discovery rate of 1%, calculated by using a reverse database strategy.

### 2.4 Protein Analysis

#### 2.4.1 Prediction of Physicochemical Parameters

The complete amino acid sequence of histones H4-W6ULY2, H2A-W6UJM4, H2A-W6U132, and H2A-W6U0N3 were obtained from UniProt. Online tools from Expasy Prot Param (Wilkins et al., 1999) were used to analyze the histones identified.

#### 2.4.2 Analysis of Histone Similarity

Similarities between histones from *E. granulosus* and other organisms were studied by BLAST-P ^3^ with the complete sequence of each histone, non-redundant database, and the organism as inputs.

#### 2.4.3 Prediction of the Secondary Structure, Domain, and Post-Translational Modification Site of the *E. granulosus* Histones Identified

Prediction analysis was performed using the following on-line analysis software: SOPMA^4^ for secondary structure, Pfam^5^, Conserved Domains tool from NCBI^6^ and Interpro^7^ for domains, and MusiteDeep^8^, DeepNitro (Xie, Y. 2018) and CKSAAP_CitrSite^9^ for post-translational modification site.

#### 2.4.4 Prediction of the Tertiary Structure of Histones

The amino acid sequence of each protein was used to search structurally homologous sequences in the Protein Data Bank, using the Sequences Annotated by Structure (SAS) (Milburn et al., 1998). Since the best templates from SAS for the sequences of histones H4 and H2A did not cover a large part of each sequence, the *Ab initio* methodology was selected. Molecular modeling was performed using *ab initio* modeling from the Robetta^10^ platform. Histone H4 was modeled by the *Ab initio* method, in which the target starts as an extended chain and the *Ab initio* Rosetta fragment assembly method folds the chain. Histones H2A were modeled by the TrRosetta method, a deep learning-based modeling method (threading). The quality of the models was analyzed by the ERRAT platform, using the Ramachandran, ERRAT and VERIFY3D options. Then, each model was validated by molecular dynamics simulation by using the Nanoscale Molecular Dynamics software. Each protein was solvated with explicit solvent by using the TIP3 water model, in a water box with the following dimensions: 79.31, 71.26, 52.77; 59.50, 66.47, 134.07; 91.28, 98.08, 144.33 and 97.56, 66.92, 82.35 (x, y, z in Å) for H4-W6ULY2, H2A-W6UJM4, H2A-W6U132 and H2A-W6U0N3, respectively. The system was neutralized with NaCl at an ionic concentration of 0.15 M. Periodic boundary conditions were used. The CHARMM36 force field was used in all molecular dynamics simulations in a standard number of particles, pressure (1 atm) and temperature. The simulation protocols involved: 2000 steps of minimization by the conjugate gradient method; 0.29 ns of heating from 60K to 300K; 1 ns equilibration maintained at 300K; and unrestrained production of 50 ns maintained at 300K, considering potential energy to confirm the thermodynamic equilibration of each molecule. The root mean-square deviation (RMSD) and root mean square fluctuation (RMSF) were calculated. Structures were analyzed and visualized with Visual Molecular Dynamics (version 1.9.3) and Molecular Operating Environment.

### 2.5 Histone Epitope Studies by Bioinformatics

The B-cell linear epitopes (Lep) of each protein were predicted using eight software programs. The results selected were those of ABCpred (score above 0.85) (Saha and Raghava, 2006), also identified in regions of at least five adjacent amino acids by Bepipred Linear Epitope Prediction 2.0 (threshold: 0.5) or by Bepipred Linear Epitope Prediction (threshold: 0.35) and by at least three of the following software programs: Chou & Fasman Beta-Turn Prediction (threshold indicated by the server for each protein sequence), Emini Surface Accessibility Prediction (threshold: 1.0), Karplus & Schulz Flexibility Prediction (threshold:1.0), Kolaskar & Tongaonkar Antigenicity (threshold indicated by the server for each protein sequence), and Parker Hydrophilicity Prediction (threshold indicated by the server for each protein sequence) in IEDB (Immune epitope database and analysis resource)^11^. The B-cell conformational epitopes (Cep) of each protein were predicted with DiscoTope^12^ 2.0, using a threshold of -3.7 (sensitivity= 0.47 and specificity= 0.75) for the final structure (50 ns - unrestricted trajectory) of each histone. This software uses a combination of amino acid statistics, spatial information, and surface exposure. It is trained on a compiled data set of discontinuous epitopes from X-ray structures of antibody/antigen protein complexes.

### 2.6 Sequencing of Genomic DNA Encoding Histone Epitopes From the *E. granulosus* G1

*E. granulosus* DNA was extracted from an *E. granulosus* G1 metacestode obtained from a cow's liver, from a slaughterhouse located in Buenos Aires, Argentina (Dr. Tatiana Aronowicz, SENASA). DNA was isolated with a lysis solution of 2% cetyltrimethyl ammonium bromide (w/v) (Stanton, Buenos Aires, Argentina), 1.4 M NaCl_2_, 20 mM EDTA (Merck Química Argentina, Buenos Aires, Argentina), 100 mM Tris-Cl (Plus one Tris, GE-Healthcare, Bio-sciences, Uppsala, Sweden), 0.175% β-mercaptoethanol (MP Illkirch- France) (v/v), pH 7.5 and then chloroform/isoamyl alcohol (24:1), precipitated with isopropyl alcohol and washed with 70% ethanol/10 mM ammonium acetate. The final DNA concentration was 730 ng/µL (260 nm, spectrophotometer, WPA BIOWAVE II+, Biochrom Ltd., Cambridge, England) and 18 ng was used for the integrity study in gel electrophoresis (1% agarose LE; PBL-Buenos Aires, Argentina) and for PCR. All primers were obtained from Gene Biotech SRL (Buenos Aires, Argentina). The genotype of the metacestode was determined by COX1C, using the primer F: 5’-CTGTTTTGGCTGCGGCTATT-3’; R: 5’-AGCCGTCTTCACATCCAACC-3’. Then, specific primers were designed to amplify an optimal fragment size between ≈250 bp-500 bp including the coding region for each predicted linear epitope with the Primer-Blast tool available in the NCBI website (Ye et al., 2012). The primer sequences are listed in **Table 1**. Each reaction tube contained: 1.5 mM MgCl2 (5x, Colorless GoTaq^®^ Reaction Buffer, Promega, Madison, WI, USA), 0.2 mM of dNTPs mix (dGTP, dCTP, dTTP and dATP, Promega, Madison, WI, USA), 1 µM forward primer, 1 µM reverse primer, 1.25 units of DNA polymerase (GoTaq^®^ polymerase, Promega, Madison, WI, USA), 18 ng of DNA template and nuclease-free water up to 50 µL (ultrapure, PB-L, Productos Bio-lógicos). The PCR protocols consisted of an initial denaturation of the template (95° C for 2 min) followed by 35 cycles of template denaturation (95° C for 1 min), annealing of primers (appropriate temperature according to pair of primers for 1 min), and DNA extension (72° C for the corresponding time according to product size); and a final extension of 72° C for 5 min, using a Mastercycler personal (Eppendorf, Hamburg, Germany). Then, 17.5 µL of product was observed by agarose electrophoresis (LE molecular biology grade, PB-L, Productos Bio-Lógicos, Buenos Aires, Argentina) using the 100-1000-bp ladder (Dongsheng Biotech Co., Ltd., Guangzhou, China). Bands were purified using the Wizard^®^ SV Gel and PCR Clean-Up System (Promega Co., USA) and sequenced in the CEDIE “Dr César Bergadá (CONICET- Hospital de Niños ‘Ricardo Gutiérrez’, Buenos Aires, Argentina). The sequences obtained were studied by BLAST and manual alignment.

**Table 1:** Primer sequences used for amplifying epitope sequences by PCR.

Epitope	Primer sequences	Product size (bp)	Tm (°C)	Extension time (seconds)
H4-W6ULY2_11-26_	F: 5’-ATGTCTGGTCGCGGTAAAGG-3’ R: 5’-ACGACCCTGGCGTTTAAGAG-3’	288	54	18
H4-W6ULY2_134-149;158-173_	F: 5’-TGGGTGCAAGTCAGTGTACC-3’ R: 5’-CGCCTCACCAGTAACTCACA-3’	227	54	14
H2A-W6UJM4_27-42_	F: 5’-TCCGTCAGTGGCAACATTCA-3’ R: 5’-GGGCGGCCTAGATGGACTTA-3’	324	56	20
H2A-W6UJM4_92-107_	F: 5’-ACACCAAGAGGTTTGGACGG-3’ R: 5’-TCATGCGTGTTTTAGGCTGG-3’	470	54	28
H2A-W6U132_175-190_	F: 5’-AGCCACCGTAAAAGCTGACA-3’ R: 5’-AATGGGCGGTAAGAAGTCCA-3’	281	52	17
H2A-W6U132_262-277_	F: 5’-GATGTCGAATGCTCACCGGA-3’ R: 5’-GCCGTGGAGAGTAGTTGGTC-3’	294	56	18
H2A-W6U0N3_123-138,138-153,170-185_	F: 5’-TGCCTAATACCACGTACGGC-3’ R: 5’-CGCTGACCAAACAGTTCAAGC-3’	342	54	21

## 3 Results

### 3.1 Characterization of *E. granulosus* Antigenic Histones

**Figure 1:**
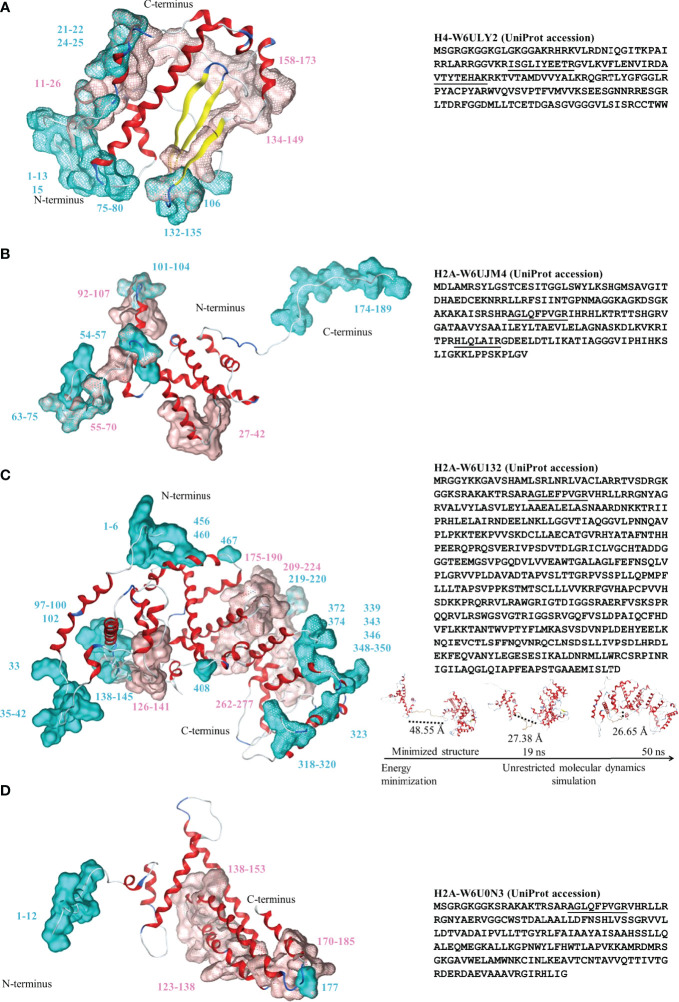
Left: Three-dimensional structure for the histones identified by proteomic analysis. **(A)** Histone H4-W6ULY2, **(B)** Histone H2A-W6UJM4, **(C)** Histone H2A-W6U132, and **(D)** Histone H2A-W6U0N3. Conformational and linear epitopes are annotated over the three-dimensional structure by cyan and pink van der Waals surfaces, respectively. The backbone 3D structure is shown in ribbons: alpha-helices (red), beta-sheet (yellow), turns (blue) and loops (light-blue). Right: the corresponding sequences in one-letter code for the four studied histones. The peptides identified by MS/MS are underlined. For the special case of H2A-W6U132 (**C**, right, bottom), snapshots of the structural conformation after the energy minimization/molecular dynamics steps are shown and dotted lines shown the loop distances between the histone and the WGR-PARP domains.

Several proteins from EGPE cells were recognized by the CE sera after performing the affinity column. Histones H4-W6ULY2, H2A-W6UJM4 and H2A-W6U132 were identified in the supernatant of EGPE colonies. The same histone H4 and histone H2A-W6U0N3 were recognized in the cellular extracts. The sequence of the histones’ peptides obtained by MS/MS are shown in **Table 2** and, the histones complete sequences are shown in **Figure 1**. Some histone sequences of *F. hepatica* are similar to those of *E. granulosus* (**Table 3**). Despite this, control sera only recognized histone H2A-W6U0N3. No protein sequences were described for histones H4 or H2A in *Taenia solium*.

**Table 2:** Histone identified by proteomic analysis: MS/MS-Data.

Protein Source	Description(UniProt Accesion)	Coverage [%]	Peptides/sequence	PSMs/TheoreticalMH+ [Da]	Unique Peptides	Theoretical MW [kDa]	Calculated pI	SequestScore Value
Colonies supernatant	Histone H4 OS=Echinococcus granulosus OX=6210 GN=EGR_10657 PE=3 SV=1 (W6ULY2)	16	3 /	3 /	3	19.3	10.48	7.72
			DAVTYTEHAK	1134.54258				
			ISGLIYEETR	1180.62083				
			VFLENVIR	989.57784				
Colonies supernatant	Histone H2A OS=Echinococcus granulosus OX=6210 GN=EGR_06849 PE=3 SV=1 (W6UJM4)	8	2 /	3 /	2	20.2	10.08	4.51
			AGLQFPVGR	944.53123				
			HLQLAIR	850.52575				
Colonies supernatant	Histone H2A OS=Echinococcus granulosus OX=6210 GN=EGR_10393 PE=3 SV=1 (W6U132)	2	1 /	1 /	1	56.6	9.45	1.76
			AGLEFPVGR	945.51524				
Cell extract	Histone H4 OS=Echinococcus granulosus OX=6210 GN=EGR_10657 PE=3 SV=1 (W6ULY2)	10	2 /	2 /	2	19.3	10.48	4.79
			ISGLIYEETR	1180.62083				
			VFLENVIR	989.57784				
Cell extract	Histone H2A OS=Echinococcus granulosus OX=6210 GN=EGR_11152 PE=3 SV=1 (W6U0N3)	5	1 /	1 /	1	21.1	10.27	1.75
			AGLQFPVGR	944.53123				

**Table 3:** Amino acid sequence similarity with *Fasciola hepatica*.

Histone	Identity (%)	Accession (NCBI)
**H4-W6ULY2 (amino acids 1-103)**	100	THD21169.1
**H2A-W6UJM4 (amino acids 59-189)**	97.71	THD 18298.1
**H2A-W6U132 (amino acids 30-149)**	86.18	THD21592.1
**H2A-W6U0N3 (amino acids 1-58)**	81.03	THD21592.1

For all the histones analyzed, the estimated half-life was 30 h in *in vitro* mammalian reticulocytes, more than 20 h in yeast and more than 10 h in *Escherichia coli*. Histones H4-W6ULY2 and H2A-W6U132 had an instability index above 40, which allows classifying the proteins as unstable, whereas histones H2A-W6UJM4 and H2A-W6U0N3 were classified as stable. The aliphatic index, the Grand average of hydropathicity (GRAVY) and the predicted secondary structure of histones are shown in **Table 4**. The predicted secondary structure of histones H2A was different: H2A-W6U0N3 had the highest content of α-helices, H2A-W6UJM4 showed the lowest number of β-turns and H2A-W6U0N3 showed the lowest proportion of random coil and highest number of extended strands. H2A-W6U132 had the same proportion of β-turns as H2A-W6U0N3 and the same proportion of random coils as H2A-W6UJM4.

**Table 4:** Physicochemical parameters and secondary structure of the histones identified.

Histone	Instability index	Aliphatic index	Hydropathicity (GRAVY)	Structure			
				α-helices	B-turns	Random coils	Extended strands
**H4-W6ULY2**	52.68	78.46	-0.371	36.00	10.86	35.43	17.71
**H2A-W6UJM4**	35.07	93.02	-0.225	42.86	6.88	38.62	11.64
**H2A-W6U132**	47.10	93.53	-0.174	38.03	10.04	37.84	14.09
**H2A-W6U0N3**	24.36	96.15	0.009	51.28	11.79	20.51	16.41

A high-quality tridimensional model was obtained for each histone according to Ramachandran plot analysis (89.9 - 97.1% of the residues in the most favored region); ERRAT (94.94 – 100% of the protein with an error value below the rejection limit) and VERIFY3D (71.24 - 84.57% of the residues with an average score 3D/1D ≥ 0.2). A molecular dynamics simulation was performed for each model. The analysis of total potential energy showed that all models reached the thermodynamic equilibrium during the equilibration step of the molecular dynamics simulation, reaching average values of -92488 ± 153, -165501 ± 192, -404888 ± 321 and -168200 ± 193 Kcal/mol over all trajectories for histones H4-W6ULY2, H2A-W6UJM4, H2A-W6U132, and H2A-W6U0N3, respectively. The analysis of the RMSD showed that the global mobility is generally associated with high RMSD rates, even when average structure is taken as reference (**Table 5** and **Supplementary Figure 2**). In addition, a graphical revision of the dynamic trajectories showed that some regions are clearly very stable in the space and have relative movements between them. This observation is confirmed by the lowered rates in the RMSD values as it is shown in the **Supplementary Figure 2** for each sequence range.

**Table 5:** Histones RMSD for the total protein and the regions.

Protein	RMSD ± SD (Minimized structure) Å	RMSD ± SD (Average structure) Å
**H4 - W6ULY2 (175 aa)**	5.57 ± 1.44	3.90 ± 0.85
**1-14**	5.18 ± 1.57	3.53 ± 0.65
**14-175**	4.00 ± 0.95	1.85 ± 0.69
**H2A - W6UJM4 (189 aa)**	8.64 ± 2.34	5.88 ± 1.57
**1-160**	4.74 ± 1.24	2.75 ± 0.65
**160-189**	5.54 ± 1.43	4.60 ± 1.04
**H2A - W6U132 (518 aa)**	12.86 ± 4.96	7.78 ± 1.50
**1-130**	6.79 ± 1.51	4.11 ± 0.83
**130-150**	8.68 ± 2.89	5.27 ± 1.43
**150-518**	5.27 ± 1.15	2.23 ± 0.73
**H2A - W6U0N3 (195 aa)**	5.41 ± 1.20	3.72 ± 0.74
**1-14**	4.13 ± 1.53	3.30 ± 1.12
**14-195**	4.25 ± 1.00	2.17 ± 0.61

However, a graphical revision of the dynamic trajectories showed that some regions are clearly very stable in the space and have relative movements between them. These histones have a tridimensional structure domain under canonical histone folding, which would function like histones, and other domains that could have other properties.

Histone H4 showed a region with more mobility, involving 64.3% of non-polar amino acids, particularly glycine among the first fourteen amino acids, and β-turns in the C-terminus. The three histones H2A had different tertiary structures. Histone H2A-W6UJM4 showed a more mobile region, a 160-189 amino acid sequence, with a high content of non-polar amino acids (66.6%), particularly glycine and proline. Histone H2A-W6U0N3 had a histone fold with a tail in the N-terminal region with high content of non-polar amino acids (50.0%), particularly glycine. Histone H2A-W6U132 showed a typical histone domain in the N-terminus, linked to a mobile region with high content of non-polar amino acids (57.1%), particularly proline. Its amino acid composition was found to be similar to the C-terminus of histone H2A (pfam16211 and IPR032454, amino acids 117-149); a second folded domain involves two domains: WGR (pfam05406 and IPR008893, amino acids 334-397 and 350-397, respectively) and PARP (pfam00644 and IPR012317, amino acids 466-516 and 410-518, respectively). **Figure 1** (insert in C) shows the different structures acquired during the molecular dynamics simulation of histone H2A-W6U132. The distance between amino acids 1-130 and 150-518 decreases by 45% between the minimized structure and the 50 ns structure.

### 3.2 Epitope Prediction

The epitopes were localized mainly in the mobile regions of the histones (**Figure 1** and the RMSF in **Supplementary Figure 3**). In the epitopes, the ubiquitination and only-methylation sites were found to be localized only in the Cep. Those for ubiquitination were found only in H2A-Cep from the supernatant in W6UJM4 (K74 and K180) and W6U132 (K42 and K145), whereas those for methylation were found in H2A-W6U132 (R2 and R33) and in H2A-W6U0N3 and H4-W6ULY2 (R4). Two putative sites for citrullination were shared with those for methylation: R4 in the Cep of H4-W6ULY2 and H2A-W6U0N3, and R33 in the Cep of H2A-W6U132. In H2A-W6U132, the only-citrullination sites were Cep sites (R343 and R350), whereas those in H2A-W6U0N3 were Cep R11 and Lep (R136, R139, R176 and R179); those in H2A-W6UJM4 were Lep R93 and Lep-Cep R104, and those in H4-W6ULY2 were Lep (R18 and R170). The putative site for phosphorylation in the intracellular H2A-W6U0N3 was only in Cep (S2), whereas those in H4-W6ULY2 were in Cep (S2) and Lep (S138) and those in H2A-W6U132 were in Lep (S184 and S272) and Cep (S348). The acetylation or methylation putative sites were shared for Lep and Cep in the same amino acid, K21 for H4-W6ULY2 and K66 for H2A-W6UJM4. The putative site for acetylation was absent in epitopes of H2A-W6U132, whereas that in the intracellular histone H2A-W6U0N3 was only in Cep (K6 and K9) and that in the supernatant of H2A-W6UJM4 was in Cep (K72) and shared K63 and K70 in Cep-Lep. H4-W6ULY2 presented more putative sites for acetylation in epitopes, in Lep K17, in Cep K6, K9 and K80 and in Lep-Cep K13. No nitration sites were found in the epitopes of these histones.

*E. granulosus* and *F. hepatica* were found to share Lep 11-26 from histone H4-W6ULY2 and Lep 92-107 from H2A-W6UJM4 but, these histones were not eluted by the control affinity column.

### 3.3 DNA Sequencing of Histone Epitope in the *E. granulosus* G1 Genome

**Figure 2:**
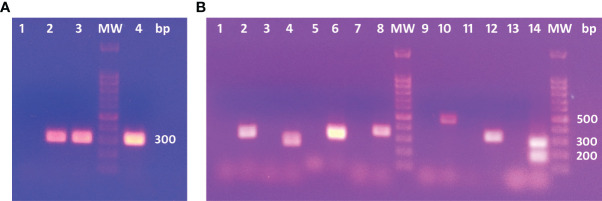
**(A)** PCR products in electrophoresis agarose gel for COX1C primers. 1: No template. 2 and 3: Positive controls. 4: Cow’s liver DNA template. **(B)** Agarose gel electrophoresis for PCR products. 1, 3, 5, 7, 9, 11 and 13: No template controls for each PCR assay. 2: H4-W6ULY2_11-26_. 4: H4-W6ULY2_134-149; 158-173_. 6: H2A-W6U132_262-277_. 8: H2A-W6U0N3_123-138,138-153,170-185_. 10: H2A-W6UJM4_92-107_. 12:H2A-W6UJM4_27-42_. 14: H2A-W6U132_175-190_. H2A-W6U132_175-190_ shows two bands: the expected product of 281 bp and an unspecific product with lower molecular weight.

The *E. granulosus* genotype was identified by COX1 analysis (**Figure 2A**). The predictive histone epitopes were amplified by PCR in DNA from a metacestode localized in a bovine liver (**Figure 2B**). The epitopes sequenced bands obtained showed 90.06 to 100% identity to the corresponding coding gene by Clustal Omega. The double band < 200 bp found in H2A-W6U132_175-190_ could not be sequenced.

## 4 Discussion

Serological diagnosis in CE lacks inter-laboratory standard, although many recombinant or synthetic proteins have been proposed to be useful. However, the recombinant or synthetic antigenic proteins assayed have less sensitivity and specificity than *ex vivo* parasite antigens and/or predictive values have not been conclusive. These are the cases of AgB (Hernández-González et al., 2008; Savardashtaki et al., 2017; Han et al., 2019; Salah et al., 2021), Ag5 (Barbieri et al., 1998), EPC1-calcium binding protein from *E. granulosus* protoscoleces (Fathi et al., 2016), and recombinant tubulins obtained, but not clinical studies with these antigens have been performed (Liu et al., 2018).

In this work, we studied several histones identified by proteomic analysis of antisera affinity of human host CE sera. Histones are proteins localized mainly in cellular nucleosomes and less frequently in the cytoplasm and extracellular space. Histone concentration in human serum is a marker of tissue damage (normal values: 0.8 ng/mL). In severe trauma, sepsis, cancer and autoimmune disease, high histone concentration and other markers predict multi-organic failure and death (Chen et al., 2014; Silk et al., 2017; Lu et al., 2020). Histones H4, H3, H2A and H2B stabilize the chromatin in the nucleosome (147 bp) and H1 and H5 are the linkers between nucleosomes. Although histones are conserved among evolution, protists have more diversity than intermediate eukaryotes. Galindo et al. (2004) described H1 genetic divergences between the Platyhelminth phyla Cestode and Trematode and, showed that *E. granulosus* has two different H1 codified by different genes, one of them like that of *Trypanosoma cruzi*. Every histone has specific physiological functions and complex regulation. Histone function regulation includes chaperones, co-chaperones, and post-translational modifications (Strahl and Allis, 2000; Hammond et al., 2017), which regulate DNA and tRNA-histone associations, causing different physiological effects.

One of the findings of the present study is that, except the reactive histone H4 found intracellularly and in the supernatant of cell colonies, H2A-W6U0N3 was localized only intracellularly and the other two H2A only in the supernatant. These findings could be attributed to the histone representativeness between the two spaces, intra- and extracellular, or to the characteristics of EGPE cells (Echeverría et al., 2010). The four histones were recognized by CE sera and the sequences of the epitopes were found in DNA from the *E. granulosus* G1 metacestode with high identity. No reactivity to H1, H3 or H2B histones was found.

Histones are released to the extracellular space by exocytosis of exosomes, or by NETosis, described in granulocytes, an ionic calcium- and PAD4 dependent mechanism with or without ROS or NADPH oxidase as initial pathway (Vorobjeva and Chernyak, 2020). Wu et al. (2019) demonstrated that a non-specific inflammatory response *via* NOD and RIP2, together with a MHC-related gene and histones, can lead to the production of antibacterial peptides. In addition, immunoglobulins with degradative capacity against histones, known as Abzymes, have been detected in HIV-infected patients and autoimmune disease (Baranova et al., 2018). In addition, Waga et al. (1987) described mouse IgG3 against histones H2A and H4 and IgG2b against H2B in DNA-histone complexes released in bovine milk. However, species-specific epitope studies have not yet been performed and the effectiveness of serological markers in the humoral response of *E. granulosus*-infected hosts has not yet been studied.

For B-cell epitope analysis we chose a software program based on recurrent neural network trained with B-cell epitopes as positive data and random peptides as negative data, with a 65.9% prediction accuracy of ABCPred for Lep epitopes (Saha and Raghava, 2006) and DiscoTope for Cep epitopes (Kringelum et al., 2012). Additionally, several software programs based on different antigen characteristics were used to select the linear sequences with a high probability to be antigenic determinants. In the literature, there are different algorithms to predict epitopes, as the recently work by Pourseif et al. (2021), who proposed to analyze the epitope prediction processing of the result by adding normalization and averaging steps. However, we chose to analyze the epitope prediction by all the above-mentioned platforms using the score of every program independently.

Among the 16 amino acids considered before, five consecutive amino acids must be included by other epitope prediction machine learning software, such as a program trained on epitopes from crystal structure or by a program combining a hidden Markov model and a propensity scale method and considering three of the following characteristics: the presence of B-turns, surface accessibility, chain flexibility, antigenicity and hydrophilicity (**Supplementary Table 1**). Conformational B-cell epitopes, which constitute approximately 90% of the B-cell epitopes, were predicted with the three-dimensional structures of histones, considering solvent accessibility, amino acid statistic and spatial information with an area under the curve performance of 0.727 (Kringelum et al., 2012).

The root mean square fluctuation indicates the residue-specific flexibility of the protein system. Most of the epitopes predicted in the histones were in regions composed of loops or turns and with high RMSF values. The flexibility of the epitopes may facilitate the conformational adaptation upon antibody binding (Karplus and Schulz, 1985; Rubinstein et al., 2008). As the histones are molecules conserved among organisms, comparison between *E. granulosus* and *F. hepatica*, another relevant organism for differential serological diagnosis, highlighted Lep H4-W6ULY2_134-149, 158-173_, H2A-W6UJM4 _27-42, 55-70_, H2A-W6U132_126-141, 175-190, 209-224, 262-277,_ and H2A-W6U0N3_123-138, 138-153, 170-185_ as unshared epitopes.

The four histones from *E. granulosus* here described are different from those from other organisms. About 58% of the sequence of histone H4-W6ULY2 has 100% identity to that from humans (NCBI: NP_001029249.1), that from *E. multilocularis* (NCBI: CDI96644.1), that from *F. hepatica* (NCBI: THD21169.1), and that from *Hymenolepsis microstoma* (NCBI: CDS25250.2). About 66% of the sequence of histone H2A-W6UJM4 has 96% identity to that from humans (NCBI: 3WAA_C). About 29% of the sequence of H2A-W6U0N3 presents 76.27% of identity to H2ATYPE1-H (NCBI: NP_542163.1), and only 22% of the sequence of histone H2A-W6U132 has 82.79% of identity to H2ATYPE2-B (NCBI: NP_778235.1). Similarities were also found in the sequences of the H2A histones of *E. granulosus*, *E. multilocularis*, *F. hepatica* and *H. microstoma*. About 29% of the sequence of H2A-W6U0N3 has 81.03-82.76% of identity to that from *E. multilocularis* (NCBI: CDS35646.1), that from *F. hepatica* (NCBI: THD21592.1) and that from *H. microstoma* (NCBI: CDS33856.1). About 23-27% of the sequence of H2A-W6U132 has 85.16-88.89% identity to that from *E. multilocularis* (NCBI: CDS36059.2), that from *F. hepatica* (NCBI: THD21592.1), and that from *H. microstoma* (NCBI: CDS33856.1). Finally, 59-69% of the sequence of H2A-W6UJM4 has 64.6-100% identity to that from *E. multilocularis* (NCBI: CDS41611.1), that from *F. hepatica* (NCBI: THD18298.1), and that from *H. microstoma* (NCBI: CDS33856.1). Similar proteins described as unnamed were found in *Hydatigera taeniaeformis*, *Dibothriocephalus latus*, *Schistocephalus solidus*, *Taenia asiatica*, *Hymenolepis diminuta*, *Rodentolepis nana*, *Mesocestoides corti*, and *Spirometra erinaceieuropaei*, and as putative histones in *Schistosoma mansoni.*

The metacestode development, from oncosphere or protoscoleces, constitutes a parasite complex biological transition involved cell proliferation, differentiation and death (Koziol and Brehm, 2015) releasing cell molecules by shedding or exosomes. Moreover, inflammatory host response increases the laminar layer micro-injury producing hydatid liquid and particulate release (Casaravilla et al., 2020) favoring the interchange between host and parasite molecules (Spotin et al., 2012), Wang et al. (2019) found histone H4 from *E. granulosus* in exosomes from CE human sera, together with α-1C and β-tubulin, whereas Fratini et al. (2020) found tubulin but not histones among parasite proteins contained in the exosomes of CE patients. Increased biosynthesis of histones H3 and H4 has been related to autophagy and increased *Drosophila* life span (Lu et al., 2021). Genotoxicity insults decrease H4 biosynthesis and stimulate degradation, decreasing DNA homologous recombination and DNA repair but, in pathogenic species such as *Candida glabrata*, a high rate of non-homologous end-joining recombination and reduction in H4 levels have been described (Kumar et al., 2020). Extracellular histones H4 and H3 could interact with endothelial cells and lymphocytes, inducing lymphocyte endothelial adhesion by a complex mechanism (Yoo et al., 2016). In the present study, histone H4-W6ULY2, found intracellularly and in the supernatant of colonies, was recognized by IgG CE sera. Among H4-W6ULY2 epitopes, Lep 11-26 was found in H4 from *E. multilocularis* by bioinformatics studies.

Histones H2A and H2B have been found to be physiologically expressed in early zebrafish embryos, decaying after 48 h post-fertilization (Wu et al., 2019), and to be involved in defense mechanisms against bacteria from diverse organisms, including mammals and shrimps (Hoeksema et al., 2016), as well as to be able to confer drug resistance (Singh et al., 2010). Regarding the three H2A here described, two were found in the supernatant (W6UJM4 and W6U132) and only one in an intracellular localization (W6U0N3).

Suspicion of CE is justified when a person from an endemic area begins to suffer allergic manifestation. Parasite or host circulating histones could be responsible for the allergic manifestations and the phosphorylation and citrullination of an epitope alter the autoantibody binding, as shown in R060 kDa, a member of the Ro/LaRNP ribonucleoprotein complex (Terzoglou et al., 2006). Histone H2A-W6U132 has three putative residues for phosphorylation in epitopes (S184 and S272, S348) and other three in non-epitope sites (S357, 360 and 515). This is the most phosphorylatable histone found by sera reactivity. It is the lengthiest one with the typical histone domain within a 1-130 amino-acid section, followed by a linker from amino acid 130 to 150 and a second folded domain from amino acid 150 to 518, which is similar to the PARP domain. The two folded domains have very different mobility, and the linker shows further evidence of bending between domains. The secondary structure of the PARP is helicoidal and some authors consider PARP as the third nucleic acid (Dabin et al., 2016). The PARP macrodomain fold is found in numerous proteins and is able to bind different ADP ribose metabolites. Overall, ADP-ribosylation seems to act as a mediator of stress response upon DNA damage, converted in the nucleus to ADP-ATP-ribose repairing DNA damage. Also, PARP modulates metabolic requirements during differentiation and responds to changes in environmental signals and metabolic milieu, facilitating the adaptation of the cell to a new situation (Simonin et al., 1993; Marjanovic et al., 2017). Although the sequences of the macroH2A1.1 and this histone do not match, the tertiary protein structure is similar. The macroH2A1.1 binds ADPribose and PARP, sharing actions and interactions on chromatin, recruits PELP1 to promoters of macroH2A1-dependent genes, and cooperates to control gene expression. PARP activity regulates nuclear receptors and mediates the expression of ATP-binding cassette transporter A1 in macrophages (Marjanovic et al., 2017). This cassette is involved in drug resistance, excluding drugs from the intracellular space.

The extracellular histone H2A-W6UJM4, found in the supernatant, has a long mobile region with a high content of non-polar amino acids and 95.87% of identity with the human H2AZ in the 121 amino acids from 60 to 180. It shares the residue 38-T of H2AZ.2 corresponding to 97 of H2A-W6UJM4, according to the sequence of human H2AZ, described by Redon et al. (2002) and Horikoshi et al. (2013). This histone is not exactly as that described by Redon et al. (2002) because of the localization of a T instead of an S, described by Bönisch et al. (2012). It could be involved in a slower histone exchange in the nucleosome (Horikoshi et al., 2013), and plays a role in chromosome segregation, embryonic stem cell differentiation, asexual reproductive cell cycle (Hanson et al., 2013) and suppression of temperature-sensitive damage (Ahamed et al., 2007), necessary for host change (36 -39°C). This histone is dispersed in the nucleus and the nucleosomes containing this histone are unstable (Redon et al., 2002). This last characteristic could explain its extracellular localization, since it may be released with unstable nucleosomes. Finally, histone H2A-W6UJM4 shares the Lep 92-107 with *E. multilocularis*.

Sites for putative ubiquitination in epitopes were found in all the histones analyzed. Only histone H2A-W6U0N3 has two sites for ubiquitination (although not in an epitope region), involved in non-homologous DNA end-joining repairs (K13 and K15) (Mattiroli et al., 2012). This histone is the H2A recognized only in intracellular protein homogenates and the ubiquitination sites are involved in DNA reparation and protein degradation (Weake and Workman, 2008), this histone would be the less representative in our experimental condition, cell colony supernatant, than intracellular localization but, it triggers humoral immune response *in vivo*. Its epitope has the putative site for phosphorylation (S2), while methylated H4 histone could join this complex (Fradet-Turcotte et al., 2013). Both histones have the putative site for methylation in the epitope (R4). EGPE cells have telomere-telomere association (Echeverría et al., 2010). When the cell cycle is accelerated, G1 and/or G0 decrease, which could generate telomere-telomere association, needing end-joining repair for the parasite survival success. *E. granulosus* metacestode grows slowly and, probably, the cell cycle acceleration does not allow the protoscoleces to sprout and mature from the proliferative layer. However, this behavior could be responsible for the multiple sterile vesicles found in hosts, as described in Avila et al. (2021). Moreover, as here described, the release of intracellular or degraded histone H2A-W6U0N3 was enough to stimulate detectable reactivity of the human immune system.

Antibodies are the answer to parasite antigen presentation to the host and may represent an “old” photograph of parasite behavior. Besides, the lack of reactivity could also have a predictive meaning. The reactivity of CE sera to the parasite histones described and analyzed in this work needs further research. The histones-antigen-specific sequences are tools to investigate the correlation between host-parasite behavior and disease stage and open a new way to investigate the specific histones involved in the evolution of the infection. Every histone plays its own role: H2A-W6U132 could be involved in the expression of ATP binding cassette transporter A1 for drug resistance, H2A-W6U0N3 could be involved in cyst sterilization, H4-W6ULY2 could be involved in parasite life span, and H2A-W6UJM4 could be involved in embryonic proliferation. Among the described histones the H4-W6ULY2 is the most robust candidate because, it is canonical and it was found in both studied localization, intracellular and extracellular, in agreement with other authors (Wang et al., 2019), it is involved in the life span of the parasite and triggers the innate and adaptive immune response.

This work mainly contributed to identifying the parasite histones recognized by CE sera, providing information about the tertiary structure and putative sites for post-translational modifications, and identifying putative singular epitopes. The epitope study of histones, or any other antigenic protein, is useful to build, in the next future, multiepitope recombinant proteins. Those which identify only one histone, could increase mainly specificity, and could be correlated with biological meaning, increasing the predictive value. Moreover, in the case of sensitivity, the next study could approach the utility of multiepitope containing the best epitopes of each antigenic protein.

## Data Availability Statement

The raw data supporting the conclusions of this article will be made available by the authors, without undue reservation.

## Ethics Statement

The studies involving human participants were reviewed and approved by Comité de Ética para la Investigación Científica y Tecnológica de la Universidad Abierta Interamericana. The patients/participants provided their written informed consent to participate in this study.

## Author Contributions

AM performed protein purification, *in silico* and data analysis, and contribute to manuscript writing. FA performed cell culture, contributed *in silico* data analysis and obtained PCR results. MV performed proteomic protein identification and analysis. AJ contribute with cell culture and biochemical experiments. MP was a director of *in silico* experiments and data analysis and contribute to manuscript writing. AF was research and work director, conceived and designed the results analysis and wrote the manuscript. All authors contributed to the article and approved the submitted version.

## Funding

This work was supported by the Fundación Iberoamericana de Estudios Superiores. Chacabuco 90, Buenos Aires, Argentina.

## Conflict of Interest

The authors declare that the research was conducted in the absence of any commercial or financial relationships that could be construed as a potential conflict of interest.

## Publisher’s Note

All claims expressed in this article are solely those of the authors and do not necessarily represent those of their affiliated organizations, or those of the publisher, the editors and the reviewers. Any product that may be evaluated in this article, or claim that may be made by its manufacturer, is not guaranteed or endorsed by the publisher.

## Footnotes

1 Dirección Nacional de Epidemiología y Análisis de la Situación de Salud. Ministerio de Salud y Desarrollo Social de la Nación (Argentina, 2019). Boletín Integrado de Vigilancia N 459, https://bancos.salud.gob.ar/sites/default/files/2020-01/boletin-integrado-vigilancia-n459.pdf. View 12/15/2021.

2 Retamozo, A.R., Agüero, F.A., Fuchs, A.G. and Maglioco, A. (2021). Serological diagnosis in hydatidosis: searching standarized antigenic support. Systematic review and the meta-analysis. Interamerican Journal of Health Sciencies 1: e89-108. https://ijhsc.com/journal/article/view/41/10. View 02/01/2022.

3
https://blast.ncbi.nlm.nih.gov/Blast.cgi?PAGE=Proteins; viewed 12/27/2021

4
https://npsa-prabi.ibcp.fr/cgi-bin/npsa_automat.pl?page=/NPSA/npsa_sopma.html; viewed 03/12/2021

5
http://pfam.xfam.org/; viewed 12/09/21

6
https://www.ncbi.nlm.nih.gov/Structure/cdd/wrpsb.cgi; viewed 12/09/21

7
http://www.ebi.ac.uk/interpro/; viewed 2/10/22

8
https://www.musite.net/; viewed 12/09/21

9 123.206.31.171/CKSAAP_CitrSite/; viewed 01/12/22

10
https://robetta.bakerlab.org/; 11/05/20

11 IEDB; Viewed 04/24/2022

12 DiscoTope; Viewed 09/01/2021
