# Supplementary figures and images for: Characterization of the B-Cell Epitopes of Echinococcus granulosus Histones H4 and H2A Recognized by Sera From Patients With Liver Cysts
Source: Front Cell Infect Microbiol. 2022 Jun 13;12:901994. doi: 10.3389/fcimb.2022.901994 (PMC9234146; doi:10.3389/fcimb.2022.901994)

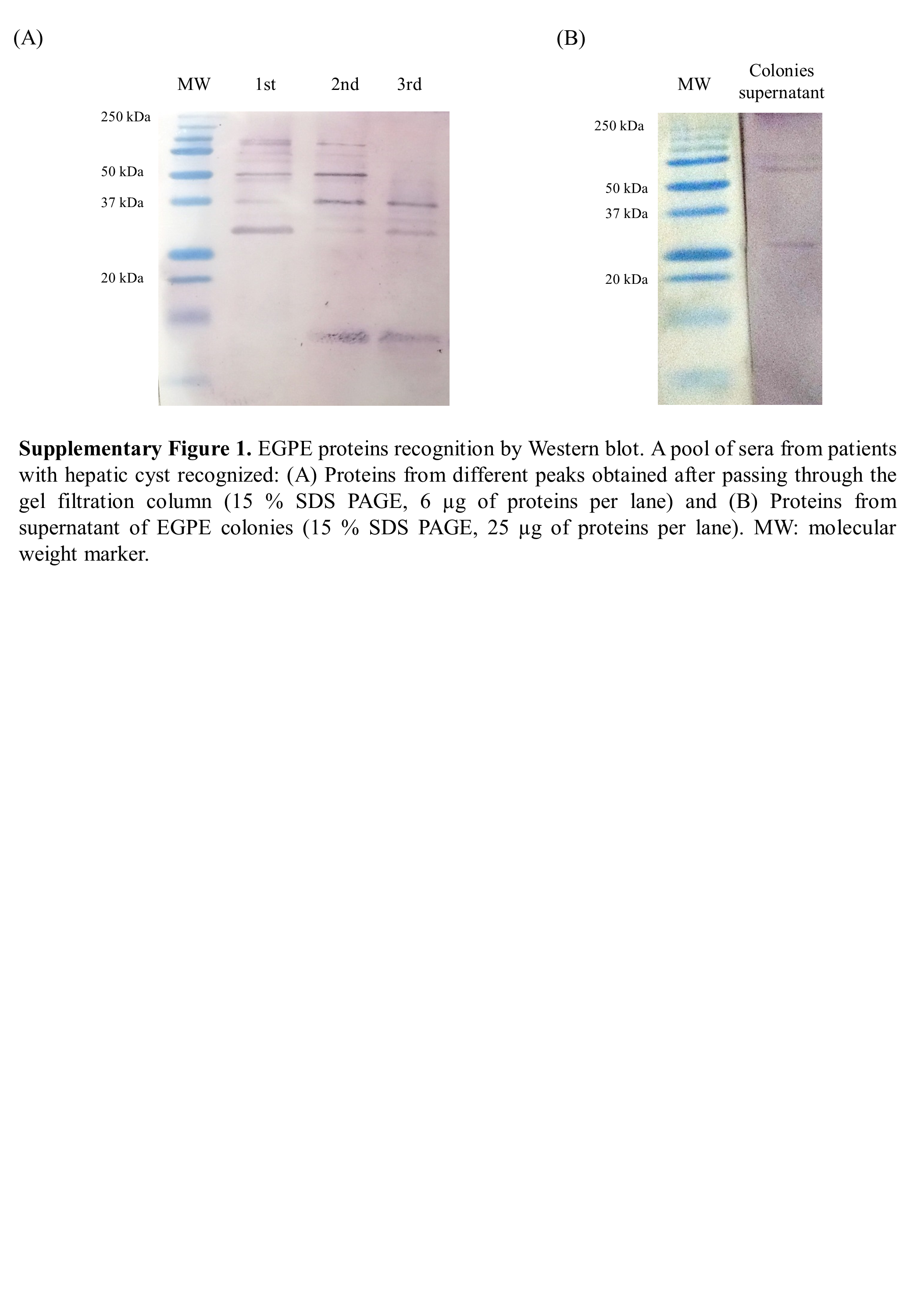

Supplement: Supplementary file 1 [file Image_1.tif]

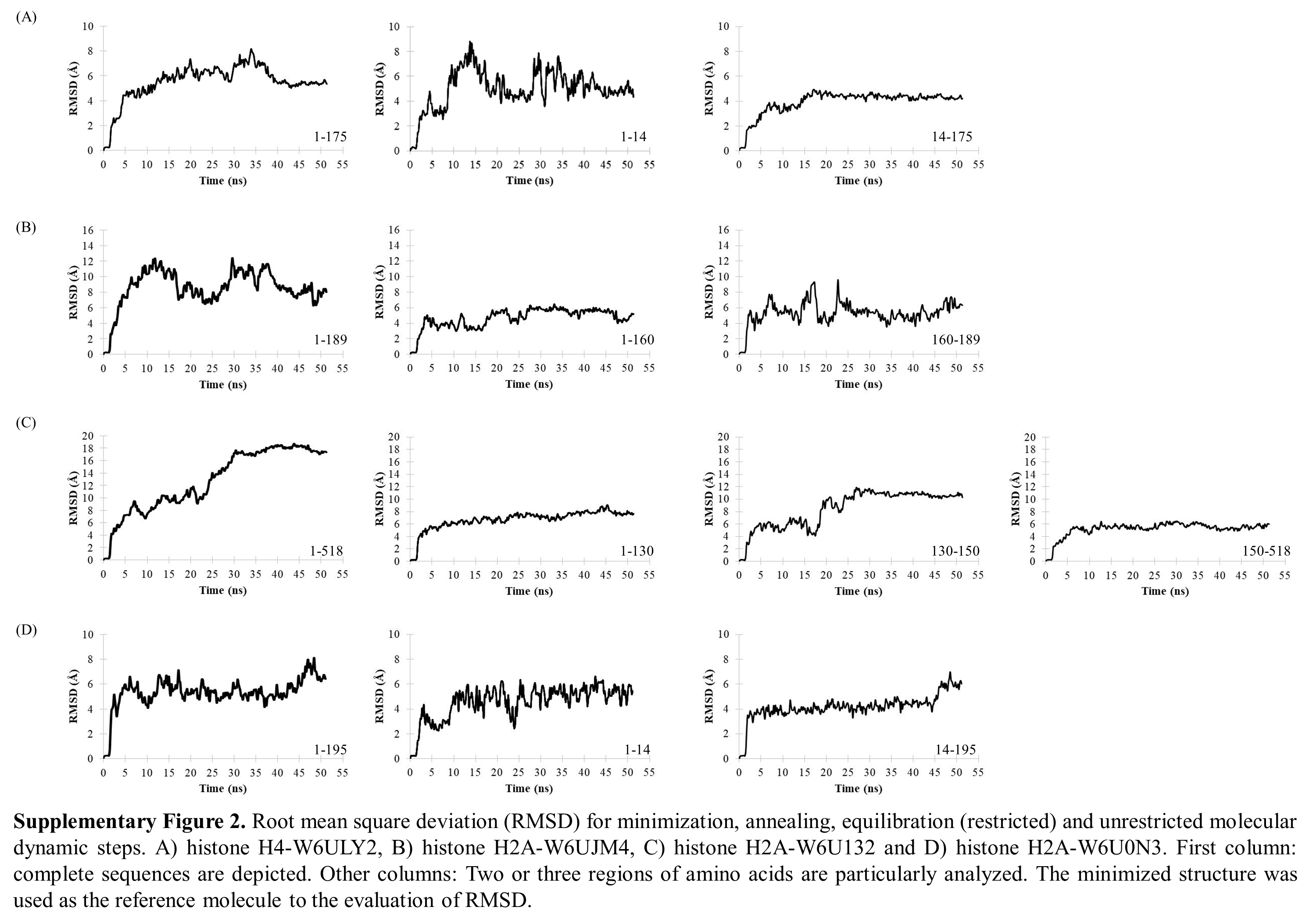

Supplement: Supplementary file 2 [file Image_2.tif]

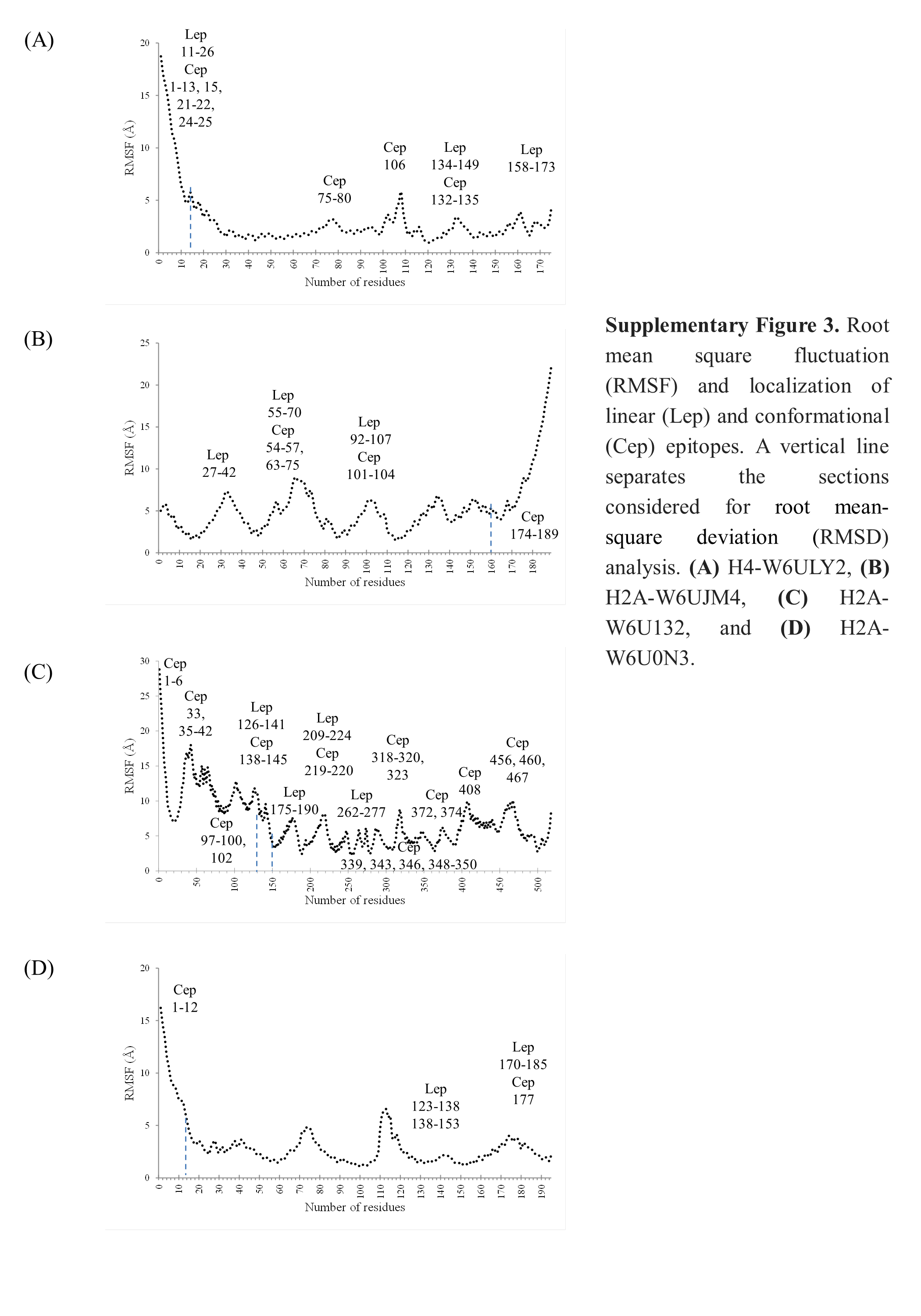

Supplement: Supplementary file 3 [file Image_3.tif]
