# Supplementary material for: Characterization of the B-Cell Epitopes of Echinococcus granulosus Histones H4 and H2A Recognized by Sera From Patients With Liver Cysts
Source: Front Cell Infect Microbiol. 2022 Jun 13;12:901994. doi: 10.3389/fcimb.2022.901994 (PMC9234146; doi:10.3389/fcimb.2022.901994)
Supplement: Supplementary file 4 [file Table_1.docx]

| **Histone** | **Epitope by ABCpred**  (Score ≥ 0.85) | **Epitope by Bepipred** (Positive score) | **Positive score**   1. Chou & Fasman Beta-Turn Prediction, 2. Emini Surface Accessibility Prediction, 3. Karplus & Schulz Flexibility Prediction, 4. Kolaskar & Tongaonkar Antigenicity 5. Parker Hydrophilicity Prediction | **Linear Epitope selected** |
| --- | --- | --- | --- | --- |
| H4  W6ULY2 | 11-26 | + | 1,2,3,5 | LGKGGAKRHRKVLRDN |
|  | 48-63 | - | 3,4 | - |
|  | 64-79 | + | 2,5 | - |
|  | 103-118 | + | 1,4 | - |
|  | 134-149 | + | 1,2,3,5 | NRRESGRLTDRFGGDM |
|  | 158-173 | + | 1,3,5 | ASGVGGGVLSISRCCT |
| H2A  W6UJM4 | 27-42 | + | 3,4,5 | HGMSAVGITDHAEDCE |
|  | 49-64 | + | 1,5 | - |
|  | 55-70 | + | 1,2,3,5 | TGPNMAGGKAGKDSGK |
|  | 92-107 | + | 1,2,3,5 | HRHLKTRTTSHGRVGA |
|  | 142-157 | - | 3,4,5 | - |
| H2A  W6U132 | 45-60 | + | 4,5 | - |
|  | 126-141 | + | 1,3,5 | VTIAQGGVLPNNQAVP |
|  | 175-190 | + | 1,2,3,5 | HPEERQPRQSVERIVP |
|  | 209-224 | + | 1,3,5 | DGGGTEEMGSVPGQDV |
|  | 229-244 | + | - | - |
|  | 262-277 | + | 1,3,4,5 | VSLTTGRPVSSPLLQP |
| H2A  W6U0N3 | 43-58 | - | 1,4,5 | - |
|  | 123-138 | + | 2,4,5 | LFHWTLAPVKKAMRDM |
|  | 138-153 | + | 1,3,5 | MRSGKGAVWELAMWNK |
|  | 170-185 | + | 1,2,3,5 | TTIVTGRDERDAEVAA |

Supplementary Table 1: Selection of linear epitopes.
